# Athlete and Hospital Worker: The Late Mr. Etherington-Smith

**Published:** 1913-04-26

**Authors:** 


### Athlete and Hospital Worker: The Late Mr. Etherington-Smith.

It may be doubted whether there was any single junior London consultant whose loss to the institutional world means as much as that of Mr. R. B. Etherington-Smith, F.R.C.S., of Bart.'s. As an oarsman, a sportsman, and a gentleman of the very best type that England can produce, he was known and beloved in wider circles than those of his profession. In his undergraduate days not many, even of his friends, suspected the popular athlete of any outstanding ability; and, indeed, rowing took up too much of his time for him to make any mark in the schools. But when he entered at Bart.'s and devoted to more serious work the energy formerly expended wholly on rowing, Etherington-Smith soon showed that he possessed talents of a very unusual order, and his progress to qualification was extraordinarily smooth and rapid. Adopting surgery as his vocation, his first appointment was on the staff of the West London Hospital, but this he resigned when his chance came at his old hospital and school. Nor did his services end with surgery. The gift of universal popularity never blinded him to the realities of life, and the unique influence of his personality was always exerted for good. He not only worked hard himself, but he set a standard of efficiency which he encouraged everyone else to strive after. As Warden of the College he took full advantage of such opportunities, and many a man in after years will achieve success which he will attribute in his inner consciousness to the example and influence of Etherington-Smith. From the published accounts it would seem that the latter operated on Tuesday of last week upon a case of gangrene of the lung, and was himself taken ill on the following day with pneumococcal peritonitis, which proved fatal in four days. Whether there was any direct connection between the two cases will probably never be known for certain, but at least it seems highly probable.

